# bioGUID: resolving, discovering, and minting identifiers for biodiversity informatics

**DOI:** 10.1186/1471-2105-10-S14-S5

**Published:** 2009-11-10

**Authors:** Roderic DM Page

**Affiliations:** 1 Division of Environmental and Evolutionary Biology, Faculty of Biomedical and Life Sciences, Graham Kerr Building, University of Glasgow, Glasgow G12 8QQ, UK

## Abstract

**Background:**

Linking together the data of interest to biodiversity researchers (including specimen records, images, taxonomic names, and DNA sequences) requires services that can mint, resolve, and discover globally unique identifiers (including, but not limited to, DOIs, HTTP URIs, and LSIDs).

**Results:**

bioGUID implements a range of services, the core ones being an OpenURL resolver for bibliographic resources, and a LSID resolver. The LSID resolver supports Linked Data-friendly resolution using HTTP 303 redirects and content negotiation. Additional services include journal ISSN look-up, author name matching, and a tool to monitor the status of biodiversity data providers.

**Conclusion:**

bioGUID is available at . Source code is available from .

## Background

One vision of biodiversity informatics is that of a cloud of digital records representing objects and events such as images, specimens, macro-molecular sequences, phenotypes, observations, publications, and taxonomic names. Each digital record would carry a globally unique identifier that would both identify that object and, given appropriate technology, be used to retrieve what we know about that object, including how it is linked to other objects. Implementing this vision, which is essentially that of Linked Data [1], requires services that can mint, resolve, and discover identifiers [2].

A **minting **service creates identifiers, and ensures their uniqueness. Given an identifier we need a **resolution **service that can retrieve the object identified (or a digital representation of that object). This service may return information in multiple formats, such as binary data (e.g., an image or a PDF document), or metadata about the object. Lastly, if we don't have an identifier for an object it should be straightforward to **discover **if one has already been minted.

**Figure 1:**
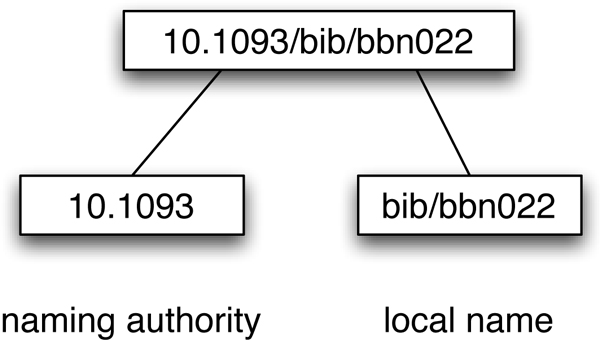
**DOI**. A DOI (10.1093/bib/bbn022) and its constituent parts. This DOI identifies reference [2].

To illustrate these services, consider Digital Object Identifiers (DOIs), widely used by the academic publishing industry to identify articles. A DOI has two parts, a naming authority assigned centrally by CrossRef [3] (and which typically corresponds to a publisher), and the local identifier, assigned by the publisher themselves (Fig. 1). Because the naming authority is assigned centrally, no two publishers will have the same naming authority, and hence no two publishers can assign the same DOI to a different article. Publishers are free to use whatever local identifier they find most useful to identify their articles, such as a primary key in a local database, or an identifier generated from metadata about the article [4]. A DOI can be resolved in a number of ways. A user can append a DOI to a HTTP resolver such as  and they will be taken to a document (typically a web page). Given a DOI one can also retrieve metadata for the corresponding article (e.g., journal and article titles, volume, page numbers) using a service provided by CrossRef,. Third-party tools can make use of this service, examples include the social bookmarking services Connotea [5] and CiteULike [6]. A user of the Connotea web site need only enter a DOI and Connotea retrieves the metadata from CrossRef, sparing the user the need to manually enter bibliographic details.

CrossRef also provides an OpenURL resolver  that takes user-supplied metadata (such as article title, journal title, volume, and pagination) and returns a DOI, if it exists. Publishers can use this service to find DOIs for articles in the "literature cited" section of an author's manuscript, and hence when the manuscript is published online it will contain electronic links to the literature cited in that manuscript.

### Minting identifiers

Ensuring an identifier is unique needs some care. Typically identifiers are unique within some scope, such as a local database, or a particular discipline. However, once one moves outside that scope, we can have unintended collisions between identifiers. As an example, the paper by Mesibov [7] contains strings that match existing GenBank accession numbers, such as DQ402119 (a human herpesvirus sequence). However, in the context of this paper, DQ402119 is a UTM grid reference for a locality in Tasmania with the co-ordinates 41° 26' 31" S, 146° 17' 02" E. Clearly, within the context of [7]DQ402119 is not intended to be interpreted as a GenBank accession number.

#### Namespaces

**Figure 2:**
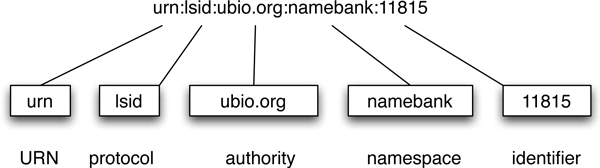
**LSID**. A LSID is prefixed with "urn:lsid", then follows the authority, namespace, and identifier components.

Collisions between identifiers can be minimised using namespaces - agreed prefixes that specify the scope within which an identifier is to be interpreted. These namespaces may be centrally assigned, for example the naming authority in a DOI, Fig. 1, or by community convention such as the Life Science Record Name (LSRN) project [8] and the "info" URI scheme [9]. The uniqueness of identifiers such as Life Science Identifiers (LSIDs) [10] and HTTP URIs is in part guaranteed by the use of Internet domain names, which are globally unique. For example, a LSID (Fig. 2) includes an authority component that is a domain name that can be resolved by the Internet DNS (typically a domain name owned by the data provider). By using the existing DNS infrastructure, LSIDs avoid the need to set up a new central naming authority.

#### Generating identifiers from metadata

One approach to minting identifiers is to generate them based on metadata for the object, which has the advantage that, in theory, two different people acting independently of each other will generate the same identifier, obviating the need for a central agency to mint the identifier. This also greatly simplifies identifier discovery - the identifier can be generated from the object at hand.

**Figure 3:**
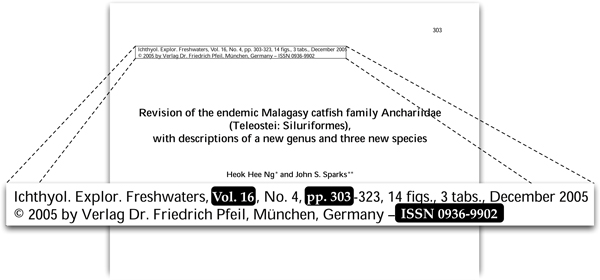
**Metadata for a published paper**. The combination of ISSN, volume number, and starting page are sufficient to uniquely identify this article. The Journal Article Citation Convention (JACC) for this article is 0936-9902:16@303.

A rather baroque example of this approach is the Serial Item Contribution Identifier (SICI) [11], which generates an identifier for a journal article from the journal's ISSN, date of publication, volume, issue, starting page, and article title, and includes various control characters and a checksum. Cameron's [4] Journal Article Citation Convention (JACC) is a rather simpler example of this approach. For a given journal article the JACC identifier is of the form <journal identifier>:<volume>@<starting page>, where typically the <journal identifier> is the ISSN of the journal. Figure 3 shows an example of a journal article and the JACC generated from information on the first page. Given that each ISSN is unique, most SICI's and JACC's will be unique (effectively the ISSN acts as the namespace for the <volume>@<starting page> part of the identifier), although complications arise if two articles start on the same page, or if a journal's pagination doesn't span a volume (for example, if issue 2 of a volume starts from page 1, as does issue 1).

#### UUIDs

Another approach to generating identifiers is to use unique strings such as UUIDs [12] which can be generated completely independently, with a very low probability that the same UUID will be generated more than once. Such a system is attractive if identifiers need to be coined independently of any central agency (for example, if one is in the field without network access and need to generate GUIDs on the fly). It may also be an issue for projects that aggregate information from a range of sources, each source of which may mint it's own GUIDs. Using UUIDs should ensure that the GUIDs are, actually, unique. The Catalogue of Life [13] adopted UUIDs for its LSIDs for this reason [14] (although the UUIDs in the 2008 release were generated centrally).

UUIDs are opaque identifiers, that is, they that contains no information about the object it identifies. In this sense it is the antithesis of an identifier such as a SICI or JACC, which embed detailed bibliographic metadata.

### Resolving identifiers

Identifiers by themselves have limited utility unless they can be resolved, that is, given an identifier we should be able to retrieve information about the object the identifier refers to. In practice, resolution means that we can retrieve information about the object from the Web. For identifiers such as HTTP URIs, this is straightforward (simply enter the URI in a web browser), but for other identifiers we need a resolution mechanism.

#### LSIDs

LSIDs are the identifier recommend by the Biodiversity Information Standards (TDWG) organisation [15]. For the biodiversity informatics community the attractions of LSIDs include the distributed nature of the identifier (no central authority is required for registering or resolving identifiers), the low cost, and the convention that resolving a LSID returns metadata in RDF. The later facilitates integrating information from multiple sources using tools being developed for the Semantic Web [16].

Despite being specifically developed to provide globally unique identifiers for objects in biological databases [10], within mainstream bioinformatics relatively few "early adopters" have deployed LSIDs [17]. In part this may because of the complexity of the resolution mechanism. A LSID client resolves a LSID in four steps:

• find location of LSID resolution service by querying the DNS service (SRV) records to find the hostname and TCP/IP service port for the LSID authority

• retrieve from the LSID authority the WSDL that defines the LSID resolution service

• retrieve a second WSDL file (the service WSDL) that specifies how the metadata and/or data corresponding to the LSID can be retrieved

• retrieve the metadata (or data), typically using HTTP GET

Not only is resolving a LSID more complicated than resolving a HTTP URI, setting up a LSID resolution service is non-trivial.

Furthermore, although LSIDs are conceptually rooted in the Semantic Web in the sense that the default metadata format is RDF, current approaches to realising the Semantic Web (such as Linked Data [1]) have settled on using HTTP URIs as the identifier. Using HTTP URIs to identify both real world objects and web pages has the potential to cause ambiguity - if I use "" as an identifier, am I talking about the city in Scotland, or the web page with that URL? The Linked Data community has adopted the use of HTTP 303 redirects and content negotiation to distinguish between a resource and a document that describes that resource [18]. A client resolving a URI  will receive a HTTP 303 ("see other") redirect, which tells the client that  identifies a non-information resource (i.e., a real-world object or concept), and it will also receive a location for a document that describes the resource (for example a web page or a RDF document). Enabling LSIDs to comply with Linked Data approaches requires a resolver that supports this mechanism.

### Discovering identifiers

The activities of minting and resolving identifiers tend to receive more attention than discovering existing identifiers. However, if a major goal of biodiversity informatics is to integrate biodiversity resources then data providers need to re-use shared global identifiers wherever possible [2], rather than simply mint new identifiers. Having multiple identifiers for the same object is potentially a major obstacle to integrating data, hence we need services that can discover whether an identifier already exists for an object. Perhaps the most obvious domain where this is relevant is literature databases, where publishers, digital repositories (e.g., JSTOR [19]), institutional archives (e.g., the Smithsonian Digital Repository [20]), indexing services (e.g., PubMed), and domain-specific databases may all assigned one or more identifiers to a scientific publication. One method the digital library community has developed to retrieve information about a bibliographic item is OpenURL [21].

#### OpenURL

**Figure 4:**
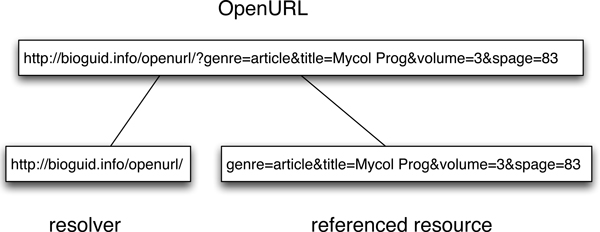
**OpenURL**. An OpenURL with two components, the resolver and the referenced resource (the later is represented as a series of key-value pairs).

As originally conceived (version 0.1), OpenURL is a HTTP GET syntax "for transporting metadata and identifiers from an information service to a linking server" [22]. Figure 4 shows a simple OpenURL that has two components, the referenced resource (in this example an article starting on page 83 of volume 3 of the journal *Mycological Progress*) and the OpenURL linking server or resolver . The OpenURL may also contain a "service identifier" (sid) that identifies the source of the OpenURL. In essence, an OpenURL is a set of key-value pairs that describe a resource. An OpenURL resolver takes this URL and returns what information (if any) it has about that resource. Whereas OpenURL 0.1 is fairly simple (and has been widely adopted), the official standard is OpenURL 1.0 [22], which has a frankly Byzantine syntax. Although the construction of an OpenURL is defined in excrutiating detail in the standards document, that standard says nothing about what the OpenURL resolver should return. Most return a web page, that is, they assume that the request is being made by a human. CrossRef is a notable exception in optionally returning XML.

One reason for the standard's complexity is its attempt to be highly generic, and thus applicable outside the library community. For example, Chute and Van de Sompel [23] use OpenURL to request regions (or metadata about regions) from a JPEG 2000 image. We could also use OpenURL to request metadata for a specimen, or indeed any other object of interest.

Some of the complexity in the OpenURL standard reflects an emphasis in the digital library community on providing the "appropriate copy" [24], for example, a copy that the user (say a member of a library) has the right to access. Most OpenURL resolvers are local in scope (e.g., they know about the contents of a particular physical library), and will return web pages telling the user if an item exists in the library (either digitally or physically). While such a service may be locally useful, if we assume that a user simply wants access to the resource (or information about the resource), and doesn't care about where it resides (i.e., "local" has no relevance when everything is "global"), then the practical utility of many OpenURL resolvers is somewhat limited.

#### COinS

**Figure 5:**
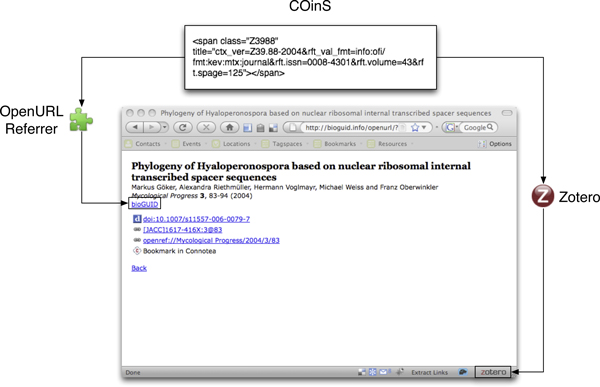
**Firefox add-ons that support OpenURL**. Two Firefox add-ons that add functionality to a web page that contains an OpenURL Context Object embedded in a <span> tag (COinS). OpenURL Referrer converts the Context Object to a clickable link to an OpenURL resolver, and Zotero extracts bibliographic metadata and adds them to a local database.

The modular nature of an OpenURL means that we can swap one OpenURL resolver for another, but keep the key-value pairs intact. OpenURL 1.0 makes this explicit with its notion of a "Context Object" (essentially the key-value pairs). The Context Object in Span (COinS) technique [25] exploits this modularity by encoding the Context Object in a <span> tag in a HTML web page so that COinS-aware tools can parse the HTML and add functionality to the web page. Examples of such tools are the Firefox browser [26] add-ons OpenURL Referrer and Zotero (Fig. 5). OpenURL Referrer [27] extracts COinS from a web page and converts them into clickable links to a user-specified OpenURL resolver. Zotero [28] extracts bibliographic metadata from the COinS to help populate the user's bibliographic database.

#### Page-level identifiers

Discovering identifiers from metadata can become challenging if the items of metadata associated with the identifier in a database differs from the metadata actually available. For example, many taxonomic citations are not to articles or books (the typical unit stored in a bibliographic database) but to an individual page. This mismatch in granularity can frustrate efforts to link identifiers for taxonomic names to identifiers for literature. Hence, it would be desirable to have a service that can return the containing document for a given page.

To illustrate, the Index Fungorum database record for *Hyaloperonospora galligena *(S. Blumer) Göker, Riethm., Voglmayr, Weiss & Oberw. 2004  gives the bibliographic source as "*Mycol. Progr. ***3**(2): 89 (2004)". There is no article in volume 3 of *Mycological Progress *that starts on page 89, so a standard search for a DOI using CrossRef's OpenURL resolver will fail. However, if we repeat the search, each time decreasing the start page by one, we will retrieve a document (Göker et al. [29]), which starts on page 83 and ends on page 94. This article contains page 89, and so we can now link the identifier for the name *Hyaloperonospora galligena *(S. Blumer) Göker, Riethm., Voglmayr, Weiss & Oberw. 2004 (urn:lsid:indexfungorum.org:name:371153) to the identifier for the publication (doi:10.1007/s11557-006-0079-7).

#### bioGUID

This paper provides a brief description of bioGUID , which implements a set of services for resolving, discovering, and minting identifiers. The initial design and development of this site is described on the bioGUID blog [30], however the version of bioGUID described in this paper differs in several ways, including support for OpenURL 1.0, and Linked Data-compliant resolution of LSIDs.

## Implementation

bioGUID is written in the PHP programming language, and the source code is available from . The LSID resolution code comes from the LSID Tester project [31]. Other third-part libraries used include the ADOdb database abstraction library [32], and the PEAR Net_DNS module [33].

DOI resolution uses CrossRef's OpenURL resolver. Article metadata is cached locally in a MySQL database to minimise requests to external services, and to facilitate locating articles based on an individual page. The MySQL database also contains metadata for articles that don't have DOIs but which are available online, such as those in DSpace repositories [34] that support OAI-PMH harvesting [35].

## Results and discussion

bioGUID  implements a range of services, the core ones being an OpenURL resolver, and a LSID resolver. Additional services include journal ISSN look-up, author name matching, and monitoring the status of biodiversity data providers.

### OpenURL resolver

**Figure 6:**
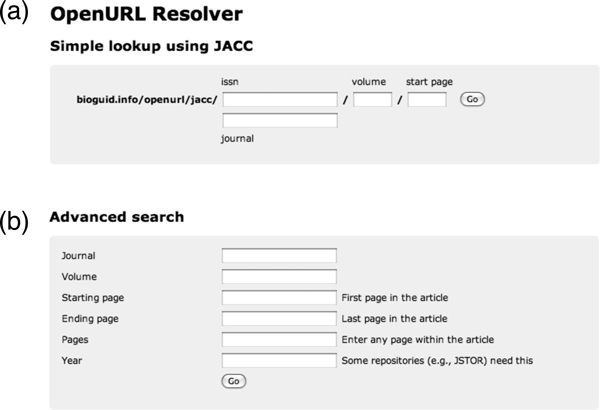
**OpenURL resolver**. Two search forms for the bioGUID OpenURL resolver for finding articles. Form (a) requires either an ISSN number of a journal or the name of the journal, togehther with the volume, and the start page of the article). Form (b) enables the user to add the year of publication, or specify an individual page within an article, rather than the start page.

The OpenURL resolver can be accessed directly by appending the OpenURL Context Object to . By default the resolver returns a HTML web page, but setting the parameter display to rdf or json instructs the resolver to return RDF or JSON, respectively, making it easy to use the resolver as a web service. If the user doesn't append the Context Object, then  displays web forms where the user can enter bibliographic metadata that describes the article (Fig. 6).

#### Minting identifiers

For journal articles the bioGUID OpenURL resolver will generate a JACC for an article, provided that sufficient metadata (journal ISSN, volume, and starting page) are available. This provides a globally unique identifier for an article, and in the absence of an existing DOI, PubMed number, or URL, it may be the only available GUID for that article.

### Resolver

bioGUID can act as a resolver for several different identifiers by appending the identifier (and it's namespace) to the base URL . For example, the JACC 1175-5326:1671@3 becomes [36], and the DOI 10.1093/bib/bbn022 becomes . For these identifiers bioGUID simply uses the Apache Web server's mod_rewrite to rewrite the URLs to OpenURLs.

#### LSIDs

**Figure 7:**
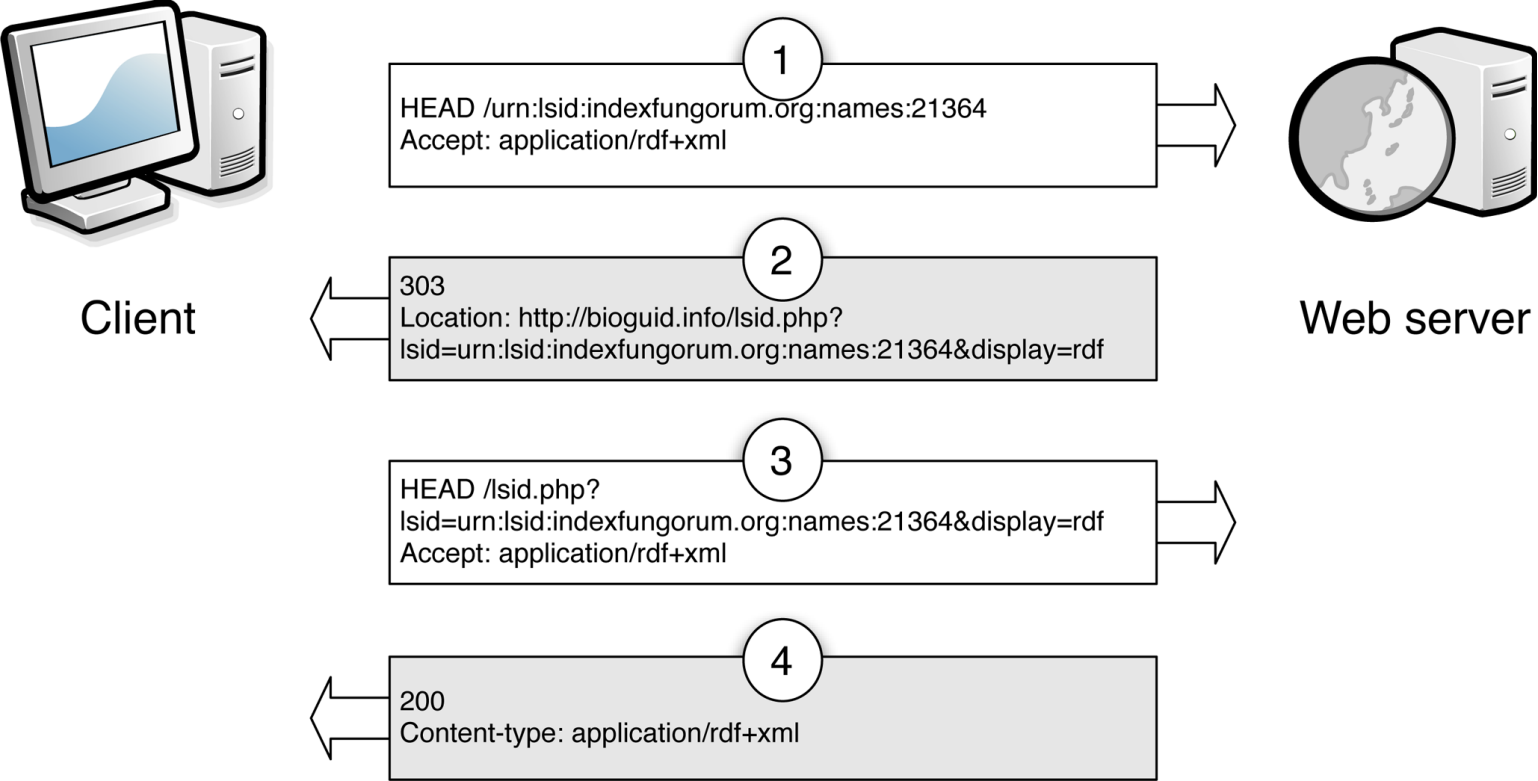
**An example of Linked Data-compliant resolution of a LSID supported by bioGUID**. In the first step (1) the client sends a request for the resource identified by the LSID, and specifies that it wants content as RDF. The bioGUID resolver returns the HTTP status code 303, and the URL where the RDF document can be found (2). The client can then request the RDF document (3), which the bioGUID web server returns (4) along with the HTTP 200 status code. Diagram is based on the output of the Vapour Linked Data validator [42].

**Figure 8:**
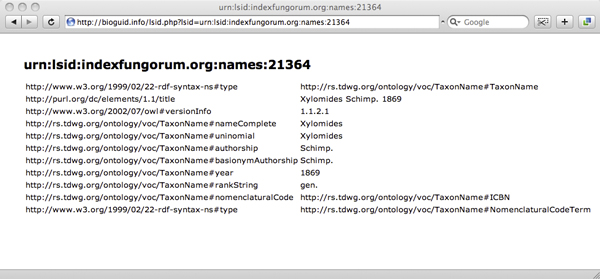
**LSID metadata in web browser**. Resolving the LSID urn:lsid:indexfungorum.org:names:21364 in a web browser results in a simple HTML display of the associated metadata.

To resolve LSIDs clients simply append the LSID to . The resolver supports HTTP 303 redirects and content negotiation [18] (Fig. 7). Clients requesting RDF will receive the raw metadata from the corresponding LSID authority, clients requesting HTML will receive a simple web page displaying a human-readable version of the metadata (Fig. 8).

### Other services

In addition to the core services listed above, bioGUID provides additional (sometimes experimental) services.

#### Journal ISSN lookup

bioGUID has a local database of journal names and ISSN numbers. A user can lookup a ISSN for a journal name by appending the journal name (or abbreviation) to the URL . This service returns a list of titles that match the request, together with their ISSNs, in JSON format. The bioGUID OpenURL resolver web page uses this service to find the ISSN of a journal the user has entered.

#### Author name matching

bioGUID's article cache includes author names, unfortunately for any given author there may be more than one way their name has been recorded in various bibliographic databases. For example, my name may be stored as "Roderic D. M. Page" or "R. D. M. Page". As a first step towards normalising author names bioGUID implements Feitelson's [37] weighted clique algorithm for finding equivalent names. This service takes a set of forenames (and initials) and returns a set of names that can regarded as equivalent. Names can be entered in a web form at , or the service can be called directly by sending a HTTP POST request to the URL  with a parameter names whose value is a list of author names (separated by the end-of-line character), and an optional parameter format with the value html or json.

#### Service availability

**Figure 9:**
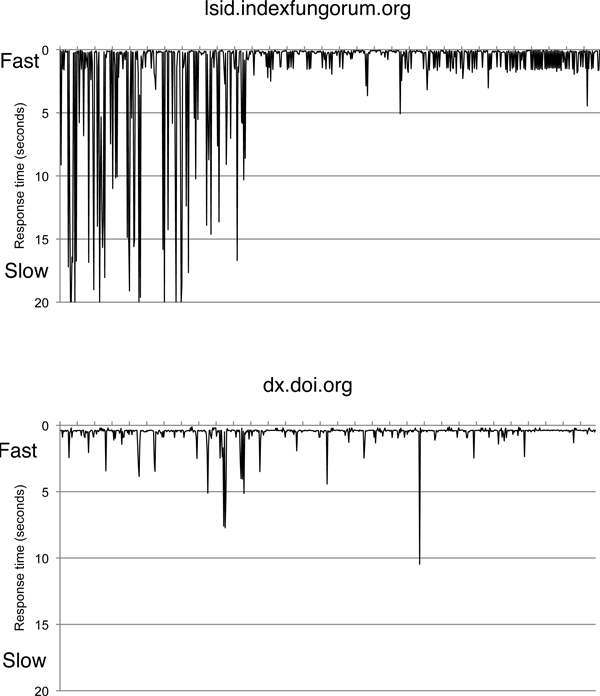
**Availability of resolution services**. Graph of the response time for the Index Fungorum LSID resolution service  and the DOI HTTP proxy  measured hourly for the month March 2009. A response time of 20 seconds indicates that the service timed out.

The vision of a linked web of biodiversity data assumes that all biodiversity data of interest is available online. Much of it isn't, but even that which is online might not always be available. A constant frustration during the course of developing bioGUID (and other projects) has the limited reliability of some data providers. Inspired by David Vieglais's The Big Dig [38],  is a simple monitoring service that every hour polls a number of web sites, LSID authorities, and DiGIR providers by sending a simple HTTP GET request and recording the HTTP status code and the time taken to respond to the request. For example, Fig. 9 shows the status of the Index Fungorum LSID authority and the DOI HTTP proxy in the month of March 2009. In the first few days of that month the Index Fungorum service frequently timed out, before becoming more stable as the month progressed. In contrast, the DOI proxy was consistently online over the same period.

## Conclusion

The lack of standard URIs for biodiversity data objects reflects a broader lack of agreement on this issue [39]. It is likely that HTTP URIs will become broadly adopted, at least outside biodiversity informatics, and they are at the heart of Linked Data [1]. However, HTTP URIs have their own problems. We are currently faced by either a great dearth of URIs or, ironically, an over abundance of them. If an entity is shared across multiple domains, then there may be multiple, competing URIs for the that entity. For example, there are numerous web sites that make statements about individual books, often using URIs that embody an ISBN. In such cases there often is not an obvious reason to choose between any of the URIs. In the same way, we have multiple identifiers for articles (such as DOIs and PubMed numbers). In such cases, tools such as OpenURL may have a role, in that the OpenURL Context Object can contain an identifier as one of its kev-value pairs. Hence, we could use the Context Object to encode this information, but delegate the choice of resolver to the client.

In cases where these is an obvious canonical source for information about an object, and that source issues HTTP URIs, it would make sense to use those URIs. Museum specimens would seem to be an obvious case (the host institution being the canonical source). However, there are few such URIs available. I regard the lack of URIs for individual specimens is one of the greatest obstacles to progress in data integration in biodiversity informatics. Again, in the absence of a recognised identifier one could adopt the OpenURL approach of encoding sufficient metadata to enable some services to retrieve a digital record about the specimen, if and when it becomes available.

bioGUID is being developed to address some of these issues, in that it supports OpenURL for literature (and experimentally for specimens), and can resolve non HTTP URI identifiers (such as DOIs and LSIDs) following Linked Data guidelines. These services can be accessed with a web browser, or programmatically. For example, the basis of my entry [40] in the Elsevier Grand Challenge [41] was a database populated by harvesting data on literature, specimens, and GenBank using bioGUID's OpenURL resolver. Having tools such as bioGUID may help mobilise biodiversity data that is currently digitised but not easily accessible, and thus bring the goal of a linked web of biodiversity data a little closer to being realised.

## Availability and requirements

• **Project Name: **bioGUID

• **Project Home Page: **. Source code is available from .

• **Operating System: **The bioGUID web site is usable with any modern web browser. The source code can be easily installed on a Mac OS X, Linux server. It has not been tested on a Windows machine.

• **Programming Language: **PHP

• **Other Requirements: **Web server

• **License: **GNU General Public License version 2

• **Any restrictions to use by non-academics: **None

## List of abbreviations used

DOI: Digital Object Identifier; DNS: Domain Name Service; GBIF: Global Biodiversity Informatics Facility; GUID: Globally Unique IDentifier; ISBN: International Standard Book Number; ISSN: International Standard Serial Number; JSON: JavaScript Object Notation; LSID: Life Science Identifier; RDF: Resource Description Format; SICI: Serial Item and Contribution Identifier; TDWG: Taxonomic Databases Working Group; URI: Uniform Resource IDentifier; URN: Uniform Resource Name; UUID: Universally Unique Identifier; WSDL: Web Service Definition Language

## Competing interests

The author declares that he has no competing interests.

## References

[B1] Linked Data - Connect Distributed Data across the Web. http://www.linkeddata.org.

[B2] Page RDM (2008). Biodiversity informatics: the challenge of linking data and the role of shared identifiers. Briefings in Bioinformatics.

[B3] CrossRef. http://www.crossref.org/.

[B4] Cameron RD (1998). Scholar-Friendly DOI Suffixes with JACC: Journal Article Citation Convention.

[B5] Connotea. http://www.connotea.org/.

[B6] CiteULike. http://www.citeulike.org/.

[B7] Mesibov R (2005). The millipede genus *Lissodesmus *Chamberlin, 1920 (Diplopoda: Polydesmida: Dalodesmidae) from Tasmania and Victoria, with descriptions of a new genus and 24 new species. Memoirs of Museum Victoria.

[B8] Life Science Record Name (LSRN). http://lsrn.org/.

[B9] "info" URI Scheme. http://info-uri.info/.

[B10] Clark T, Martin S, Liefeld T (2004). Globally distributed object identification for biological knowledgebases. Briefings in Bioinformatics.

[B11] NISO (1996). Serial Item and Contribution Identifier (SICI) ANSI/NISO Z3956-1996 (Version 2).

[B12] Leach P, Mealling M, Salz R (2005). RFC 4122: A Universally Unique Identifier (UUID) URN Namespace. ftp://ftp.rfc-editor.org/in-notes/rfc4122.txt.

[B13] Catalogue of Life. http://www.catalogueoflife.org/.

[B14] Ewen R, Orme ACJ, White RJ (2008). LSID Deployment in the Catalogue of Life. BNCOD 2008 Workshop: "Biodiversity Informatics: challenges in modelling and managing biodiversity knowledge".

[B15] Biodiversity Information Standards (TDWG). http://www.tdwg.org.

[B16] Page RDM (2006). Taxonomic names, metadata, and the Semantic Web. Biodiversity Informatics.

[B17] Martin S, Hohman MM, Liefeld T (2005). The impact of Life Science Identifier on informatics data. Drug Discovery Today.

[B18] Cool URIs for the Semantic Web. http://www.w3.org/TR/cooluris/.

[B19] JSTOR. http://www.jstor.org.

[B20] Smithsonian Digital Repository. http://si-pddr.si.edu/dspace/.

[B21] Apps A, MacIntyre R (2006). Why OpenURL?. D-Lib Magazine.

[B22] (2005). The OpenURL Framework for Context-Sensitive Services. ANSI/NISO Standard Z3988-2004.

[B23] Chute R, de Sompel HV (2008). Introducing djatoka: A Reuse Friendly, Open Source JPEG 2000 Image Server. D-Lib Magazine.

[B24] Beit-Arie O, Blake M, Caplan P, Flecker D, Ingoldsby T, Lannom LW, Mischo WH, Pentz E, Rogers S, Sompel HVD (2001). Linking to the Appropriate Copy. D-Lib Magazine.

[B25] OpenURL ContextObject in SPAN (COinS). http://ocoins.info/.

[B26] Firefox. http://www.mozilla.com/firefox/.

[B27] OpenURL Referrer. https://addons.mozilla.org/en-US/firefox/addon/4150.

[B28] Zotero. http://www.zotero.org/.

[B29] Göker M, Riethmüller A, Voglmayr H, Weiss M, Oberwinkler F (2004). Phylogeny of Hyaloperonospora based on nuclear ribosomal internal transcribed spacer sequences. Mycological Progress.

[B30] bioGUID blog. http://bioguid.blogspot.com/.

[B31] Page RDM (2008). LSID Tester, a tool for testing Life Science Identifier resolution services. Source Code for Biology and Medicine.

[B32] ADOdb Database Abstraction Library for PHP (and Python). http://adodb.sourceforge.net/.

[B33] PEAR Net_DNS. http://pear.php.net/package/Net_DNS.

[B34] Smith M, Barton M, Branschofsky M, Mcclellan G, Walker JH, Bass M, Stuve D, Tansley R (2003). DSpace: An Open Source Dynamic Digital Repository. D-Lib Magazine.

[B35] Open Archives Initiative Protocol for Metadata Harvesting (OAI-PMH). http://www.openarchives.org/pmh/.

[B36] Pyle RL, Earle JL, Greene BD (2008). Five new species of the damselfish genus *Chromis *(Perciformes: Labroidei: Pomacentridae) from deep coral reefs in the tropical western Pacific. Zootaxa.

[B37] Feitelson DG (2004). On identifying name equivalences in digital libraries. Information Research.

[B38] The Big Dig. http://bigdig.ecoforge.net/.

[B39] Vandervalk BP, McCarthy EL, Wilkinson MD (2009). Moby and Moby 2: Creatures of the Deep (Web). Briefings in Bioinformatics.

[B40] Page RDM (2008). Visualising a scientfic article.

[B41] The Elsevier Grand Challenge: Knowledge Enhancement in the Life Sciences. http://www.elseviergrandchallenge.com/.

[B42] Vapour, a Linked Data validator. http://validator.linkeddata.org/vapour.

